# Unilateral foot drop due to prone positioning in COVID‐19: A case report

**DOI:** 10.1002/ccr3.8287

**Published:** 2023-12-09

**Authors:** Harkesh Arora, Anna Bode, Sreekant Avula, Roopa Naik, Sujith Palleti, Deepak Chandramohan

**Affiliations:** ^1^ Lovelace Medical Center Albuquerque New Mexico USA; ^2^ Burrell College of Osteopathic Medicine Las Cruces New Mexico USA; ^3^ University of Minnesota, Twin Cities Minneapolis Minnesota USA; ^4^ Geisinger Wyoming Valley Medical Center Wilkes‐Barre Pennsylvania USA; ^5^ Louisiana State University Shreveport Louisiana USA; ^6^ University of Alabama at Birmingham Birmingham Alabama USA

**Keywords:** complications, compressive neuropathy, COVID‐19, foot drop, mechanical ventilation, prone positioning

## Abstract

**Key Clinical Message:**

Understanding the complications arising from prone positioning following mechanical ventilation during management of acute respiratory distress from COVID‐19.

**Abstract:**

Acute respiratory distress syndrome (ARDS) resulting from coronavirus disease 2019 (COVID‐19) has been one of the well‐known complications of the disease since it was first reported in 2020. Mechanical ventilation for severe ARDS has been widely utilized for the management of such patients. Prone positioning (PP) is associated with improved oxygenation and overall outcomes in both intubated and non‐intubated patients. However, there are several complications associated with this procedure, including compressive neuropathies. In this article, we report a case of unilateral foot drop following mechanical ventilation and PP during the management of ARDS from COVID‐19.

## 1 INTRODUCTION

COVID‐19, caused by the novel coronavirus SARS‐CoV‐2, has been associated with a myriad of complications. The most devastating of them is ARDS, with prolonged hospitalization and deaths. ARDS is diagnosed in about 33% of hospitalized COVID‐19 patients^1^ and reported in about 10.8% of all COVID‐19‐related deaths.^2^ Early PP has been noted to improve oxygenation and overall outcomes in patients with moderate to severe ARDS needing mechanical ventilation^3^ and is associated with a lower incidence of intubation.^4^, ^5^

Complications due to prolonged PP have been reported, including endotracheal tube dislodgement, temporary desaturations, brachial plexus injuries, decubitus ulcers, ischemic optic neuropathy, and compressive neuropathies.^6^, ^7^ Foot drop results from weakness of the dorsiflexors, resulting in an inability to lift the forefoot. The deep peroneal (fibular) nerve innervates the tibialis anterior muscle, which is the main dorsiflexor of the ankle. Damage to either the common peroneal (fibular) nerve or the deep peroneal (fibular) nerve can result in weakness of the tibialis anterior muscle, causing an inability to dorsiflex and resulting in the clinical presentation of foot drop.^8^

We present a patient who developed unilateral foot drop due to PP during mechanical ventilation for the management of ARDS secondary to COVID‐19 infection.

## 2 CASE DESCRIPTION

A 46‐year‐old male, unvaccinated for COVID‐19, with a past medical history of obesity (Body Mass Index 36), uncontrolled type 2 diabetes mellitus, tobacco use, and hyperlipidemia presented to the emergency room with worsening shortness of breath over a 2‐week period and reportedly tested positive for COVID‐19 in the outpatient setting. At the time of initial evaluation, the patient's room air saturation was 70%, with a respiratory rate of 36 breaths per minute requiring 10 liters of oxygen via a non‐rebreather mask, with improvement in the saturation up to 80%. The patient continued to be in respiratory distress, requiring a nasopharyngeal airway, and was admitted to the intensive care unit (ICU) for management of acute respiratory failure secondary to ARDS from the COVID‐19 infection. He was also noted to be in diabetic ketoacidosis (DKA) at the time of admission. Pertinent laboratories included serum bicarbonate <5, anion gap greater than 30, pH of 7.0, serum potassium 5.0, WBC 18.7, positive urine, and serum ketones and a hemoglobin A1C of 9.2%. Management included insulin infusion, bicarbonate infusion, midazolam and fentanyl for sedation, intravenous piperacillin‐tazobactum, dexamethasone, IV fluids, rocuronium infusion, and mechanical ventilation with a lung protective strategy and low tidal volume. PP was also utilized multiple times a day to improve oxygenation. Standard PP protocol included full patient assessment including vital signs, equipment security and positioning for endotracheal tube, chest tubes, catheters, ostomies, dressings, oral care, bed preparation with low air mattress and positioning of arms and lower extremities. Proper patient wrapping and padding techniques were followed. Tilting the patient into reverse Trendelenburg, slight, intermittent lateral repositioning (20 to 30°), and changing sides at least every 2 h was followed. He was extubated after 4 days and transferred to the medical floor. The patient was noted to exhibit an isolated foot drop. There was also significant neuropathy and loss of protective sensations in bilateral feet, which were thought to be secondary to diabetes. This led to range‐of‐motion and transfer difficulties in ADLs. He was diagnosed with unilateral foot drop due to peroneal nerve injury and was evaluated by a physical therapist. The patient was discharged with an ankle foot orthosis brace (AFO brace), a front‐wheeled walker, and outpatient follow‐up with neurology for electromyography (EMG) and physical therapy.

## 3 DISCUSSION

Peripheral nerve injuries (PNIs) are recognized as an uncommon complication of general anesthesia, with a <1% incidence in the general surgical population,^9^ and are rarely associated with prone positioning in the management of ARDS.^10^ Focal PNIs in critically ill patients have been reported, mostly in association with ICU‐acquired weakness which can result in a combination of myopathy and polyneuropathy,^11^ leading to a missed diagnosis of nerve injuries.

Peripheral nerve injuries in the setting of COVID‐19 comprise neuropathy due to inflammation from the infection, PP compression or stretch of the nerve, prolonged immobilization, nerve entrapment secondary to a hematoma, or some systemic disorder or process, including but not limited to autoimmune causes such as Guillain–Barre syndrome or critical illness polyneuropathy or myopathy.^8^, ^12^ Peripheral neuropathy can also be a sequela of diabetes mellitus (DM). Our patient had a history of type 2 DM. Peripheral neuropathy in diabetes typically develops as a chronic issue in a “stocking and glove” distribution involving the distal bilateral extremities.^13^ The acute onset and distribution of our patient's foot drop suggest diabetes was not the cause, although diabetes can be a risk factor for the development of compression neuropathy and may have contributed to the development of our patient's foot drop.^8^, ^14^ Other potential etiologies, such as Guillain–Barre syndrome, were considered but deemed unlikely as the presentation would be bilateral and not be limited to one area.^15^

The Prone Positioning in Severe Acute Respiratory Distress Syndrome (PROSEVA) trial did not include nerve injuries as complications. It could be challenging to distinguish nerve injuries from critical care weakness during the early stages.^3^ Nevertheless, there has been an increasing trend in peripheral nerve injuries secondary to prone positioning in recent years. These injuries are more common in the upper extremities, and the prevalence of diabetes mellitus, obesity, and advanced age is at higher risk. COVID‐19 causes a state of hyperinflammation, which could render peripheral nerves more susceptible to injuries. This occurs due to an imbalance in the network of proinflammatory cytokines, D‐dimer, and antibodies.^10^ Our patient had diabetes, an increased BMI, and required prolonged prone positioning; each of these is recognized as a risk factor for the development of peripheral nerve neuropathy. Prone positioning is an essential strategy to improve oxygenation. The protocols involving PP differ among institutions. Still, there should be an emphasis on changing patient positioning at frequent intervals and the involvement of physiotherapists to assess the need for early mobilization to prevent peripheral nerve injuries and other complications.

The most common form of compressive neuropathy in the lower extremities is peroneal nerve palsy.^15^ Peroneal nerve entrapment is frequently brought on by external compression of the peroneal nerve, followed by anesthesia, casting, tight bracing, compression wrapping, prolonged hospitalization, and the use of pneumatic compression devices.

After an extensive review of the existing literature, to the best of our knowledge, this is the first case report where our patient's development of left foot drop was due to nerve compression, or stretch, during PP utilized while on mechanical ventilation for the management of COVID‐19‐associated ARDS. In many cases, clinical improvement in foot drop was noted following rehabilitation. To prevent or reduce peripheral nerve injury due to prone positioning, careful positioning of patients and frequent but careful position changes should be considered.

## 4 CONCLUSION AND LEARNING POINTS FOR CLINICIANS

Peripheral nerve injuries, resulting in complications such as foot drop, have been widely reported as one of the complications of PP during mechanical ventilation in the management of COVID‐19‐associated ARDS. In patients with confounding comorbidities such as DM, careful timeline of the events leading up to the foot drop must be identified, and strategies to prevent complications for peripheral nerve injuries, including careful positioning of patients, cushioning, and changing of position at shorter intervals, should be strongly considered. Subtle changes to prone positioning protocols could help prevent the development of compressive neuropathy in COVID‐19 patients undergoing PP. In patients needing mechanical ventilation with PP for the treatment of ARDS from COVID‐19, the physicians must be vigilant in recognizing complications such as peripheral nerve compressions, leading to foot drop.

## AUTHOR CONTRIBUTIONS

**Harkesh Arora:** Conceptualization; investigation; project administration; resources; software; supervision; validation; writing – original draft; writing – review and editing. **Anna Bode:** Conceptualization; writing – original draft. **Sreekant Avula:** Formal analysis; funding acquisition; writing – review and editing. **Roopa Naik:** Investigation; resources; writing – review and editing. **Sujith Palleti:** Writing – review and editing. **Deepak Chandramohan:** Writing – review and editing.

## FUNDING INFORMATION

This research was not funded.

## CONFLICT OF INTEREST STATEMENT

The authors declare no conflict of interest.

## ETHICS STATEMENT

This is a case report that does not require formal ethical committee approval. The data were anonymously registered in our database.

## CONSENT

Written informed consent was obtained from the patient to publish this report in accordance with the journal's patient consent policy. A copy of the written consent is available for review by the editor in chief of this journal on request.

## CLINICAL TRIAL REGISTRATION

This is not an interventional study. We only reported the patient's findings from our database as a case report.

## Data Availability

Data sharing is not applicable to this article as no new data were created or analyzed in this study.
